# Supplementary material for: A Model to Predict Upstaging to Invasive Carcinoma in Patients Preoperatively Diagnosed with Low-Grade Ductal Carcinoma In Situ of the Breast
Source: Cancers (Basel). 2022 Jan 12;14(2):370. doi: 10.3390/cancers14020370 (PMC8773816; doi:10.3390/cancers14020370)
Supplement: Supplementary file 1 [file cancers-14-00370-s001.zip › cancers-1542537-supplementary.pdf]

Table S1. Association between variables and upstage (implying change of therapy). Results from univariate and multivariate logistic regression analyses.

Variable	Level	Upstage/Tot (%)	Univariate analysis			Multivariate analysis [1]		
			OR	95% CI	P-value	OR	95% CI	P-value
**Overall**	**-**	**53/295 (18.0)**	**-**	**-**	**-**	**-**	**-**	**-**
**Age at Mammotome biopsy**	+1 year		0.96	0.92-0.99	0.021	0.96	0.92-1.00	0.067
**Biopsy needle**	8G + 7G	13/45 (28.9)	Ref.	-	-	Ref.	-	-
	11G + 10G	40/245 (16.3)	0.48	0.23-0.99	0.048	0.67	0.29-1.54	0.34
	Missing	0/5						
**Post biopsy residual disease**	No	4/128 (3.1)	Ref.	-	-	Ref.	-	-
	Yes	49/167 (29.3)	12.9	4.50-36.8	<0.001	9.40	2.58-34.2	<0.001
**Post biopsy residual lesion size**	+1×$\log_2$(mm)		2.00	1.50-2.66	<0.001	1.02	0.67-1.55	0.93
**Number of cores**	+1		0.99	0.93-1.05	0.65	0.98	0.91-1.04	0.45
**Disease only in cores with microcalcifications**	No	36/132 (27.3)	Ref.	-	-	Ref.	-	-
	Yes	13/138 (9.4)	0.28	0.14-0.55	<0.001	0.38	0.18-0.80	0.010
	Missing	4/25						
